# TNF-**α** represses fibroblast to myofibroblast transition through the histone methyltransferase Setdb2

**DOI:** 10.1172/jci.insight.190836

**Published:** 2025-11-24

**Authors:** Tyler M. Bauer, Kevin D. Mangum, Samuel D. Buckley, James Shadiow, Amrita D. Joshi, Christopher O. Audu, Jadie Y. Moon, Lindsey D. Hughes, Rachel Bogel, Lam C. Tsoi, Qinmennge Li, He Zhang, Steven Kunkel, Johann E. Gudjonsson, Frank M. Davis, Katherine A. Gallagher

**Affiliations:** 1 Department of Surgery,; 2 Department of Dermatology,; 3 Department of Pathology, and; 4 Department of Microbiology and Immunology, University of Michigan, Ann Arbor, Michigan, USA.

**Keywords:** Dermatology, Immunology, Diabetes, Epigenetics

## Abstract

Fibroblast to myofibroblast transition is a critical event required for effective tissue repair. In pathologic wound repair processes, such as type 2 diabetes (T2D), fibroblast to myofibroblast transition is impaired. The exact factors that control this transition in wounds are unclear. Here, using human tissue and murine transgenic models, we show that the histone methyltransferase SETDB2 is elevated in diabetic wound fibroblasts and TNF-α represses fibroblast to myofibroblast transition via *Setdb2*. We identified that TNF-α increases *Setdb2* in fibroblasts via a JAK1,3/STAT3 signaling pathway, where pharmacologic or genetic manipulation of this pathway altered *Setdb2* in fibroblasts. We also found that fibroblasts treated with pro-inflammatory macrophage supernatants displayed increased *Setdb2* and downregulated myofibroblast genes; inhibition of the TNF-α receptor reduced the upregulation of *Setdb2*. In diabetes, we showed that TNF-α signaling was increased in wound fibroblasts, which functions to increase *Setdb2* expression and represses fibroblast to myofibroblast transition. Fibroblast-specific knockdown of SETDB2 and therapeutic inhibition of JAK1,3/STAT3 improved diabetic wound repair, where wound fibroblasts expressed increased myofibroblast genes. This study is the first to our knowledge to identify an epigenetic mechanism for reduced fibroblast to myofibroblast transition in diabetic wounds. Therapeutic targeting of the TNF-α/STAT3/SETDB2 axis in wound fibroblasts may improve diabetic wound healing.

## Abstract 2

*Setdb2* is increased in fibroblasts from diabetic wounds, and represses fibroblast to myofibroblast transition.

## Graphical abstract

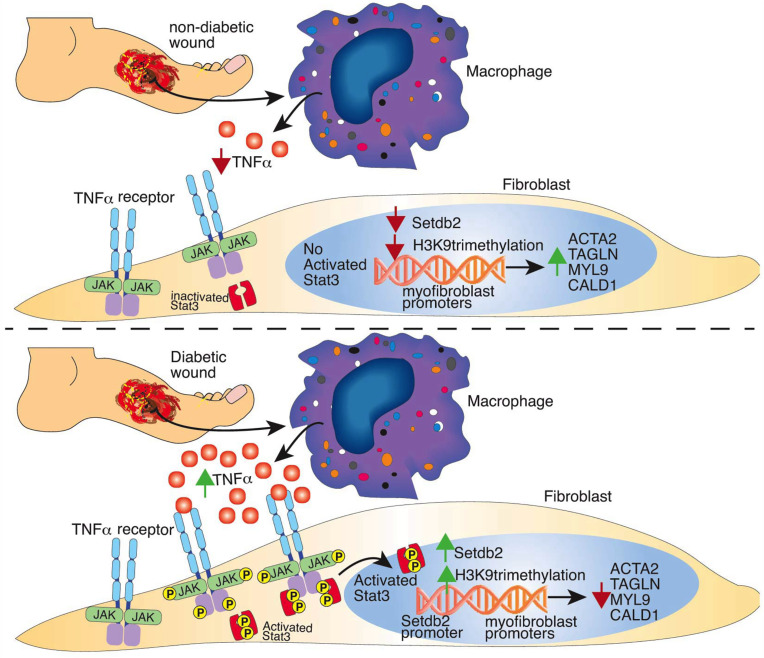

## Introduction

Successful wound repair is a tightly regulated process consisting of overlapping phases of coagulation, inflammation, proliferation, and remodeling (1). Immune and structural cells are involved in all of these phases of repair, and their interactions are essential for proper healing. Fibroblasts are a key structural cell in the dermis that carry out a number of critical functions, mainly during the proliferative and remodeling phases. Historically, fibroblasts and other structural cells have been thought of as inert and homogeneous; however, recent research has now identified profound heterogeneity in gene expression and functions of fibroblasts, even within a single tissue (2, 3). Indeed, contemporary understanding of fibroblast populations in wound healing defines specific roles, including the production of components of the extracellular matrix, contraction of wounds, breakdown of fibrin clots, and contributions to pro-inflammatory and proangiogenic functions (4, 5). While it is known that fibroblasts perform many functions that are necessary for effective tissue repair following injury, there remain significant gaps in knowledge in understanding the phenotype and cellular functions of each individual fibroblast subtype (6). Recently, single-cell multiomic approaches have augmented traditional immunohistochemistry and flow cytometry, which may lead to further advances in understanding the cellular functions of each fibroblast subtype (7–10).

Perhaps the most well-established role of fibroblasts in wound repair is the transition of fibroblasts to myofibroblasts, which serve to contract and close wounds (11). Quiescent, mature fibroblasts do not express α-smooth muscle actin (*Acta2*) to a high degree, and hence the upregulation of *Acta2* expression in fibroblasts is a marker that signifies fibroblast to myofibroblast transition (12, 13). In conditions where tissue repair is dysregulated (i.e., type 2 diabetes, T2D), reduced numbers and impaired function of myofibroblasts have been identified, signifying their importance in wound repair (14–16). The mechanisms regulating fibroblast to myofibroblast transition in wound healing remain poorly understood. Our group and others have shown that epigenetic regulation of gene expression plays a major role in defining the phenotype of immune cells in both normal wound healing and pathologic conditions (17–20). While the epigenetic regulation of immune cell phenotypes in wound healing has been relatively well studied, epigenetic control of fibroblast plasticity during normal wound healing or the diabetic state remain largely unexplored (19, 21–23).

Here, using both human tissue and murine transgenic models, we defined an epigenetic mechanism that controls fibroblast to myofibroblast transition in wound healing. We showed that the pro-inflammatory cytokine, TNF-α, is elevated in diabetic wound tissue and acts to repress fibroblast to myofibroblast transition through repression of myofibroblast gene expression via the chromatin modifying enzyme (CME) SETDB2. Translationally, using human single-cell RNA sequencing (scRNA-Seq), we found that *Setdb2* expression was increased in human T2D wounds and identified a strong inverse correlation between *Setdb2* expression and myofibroblast genes in human wound fibroblasts. Transgenic mice with a fibroblast-specific deletion of *Setdb2*, *Setdb2^fl/fl^* *ColCreERT^fl/fl^*, expressed higher levels of myofibroblast genes in wound fibroblasts. Next, since TNF-α signaling resulted in increased *Setdb2* and TNF-α signals via JAK1,3/STAT, we examined this pathway and its role in *Setdb2* expression in fibroblasts. We found that TNF-α increased phosphorylated (p-) STAT3 and that pharmacologic inhibition of JAK1,3 or genetic deletion of *Stat3* led to decreased *Setdb2* expression in wound fibroblasts. We found that supernatants from pro-inflammatory macrophages increased *Setdb2* expression in fibroblasts and repressed myofibroblast genes, and inhibition of the TNF-α receptor reduced the increase in expression of *Setdb2*, suggesting macrophage-fibroblast crosstalk in wounds may have an influence on the fibroblast to myofibroblast transition. Next, using human scRNA-Seq comparing T2D and non-T2D wounds, we found that TNF-α signaling and *Setdb2* expression were increased in T2D wound fibroblasts compared with non-T2D fibroblasts, which correlates with the reduced myofibroblast phenotype found in T2D wounds. In our murine model of T2D wound healing, both fibroblast-specific SETDB2 knockdown and injection of a commercially available, FDA-approved inhibitor of JAK1,3 improved wound healing and increased myofibroblast gene expression in fibroblasts. Our findings have therapeutic implications for improving fibroblast to myofibroblast transition in T2D wounds.

## Results

### TNF-α represses myofibroblast gene expression and increases Setdb2 expression in wound fibroblasts.

**Figure 1:**
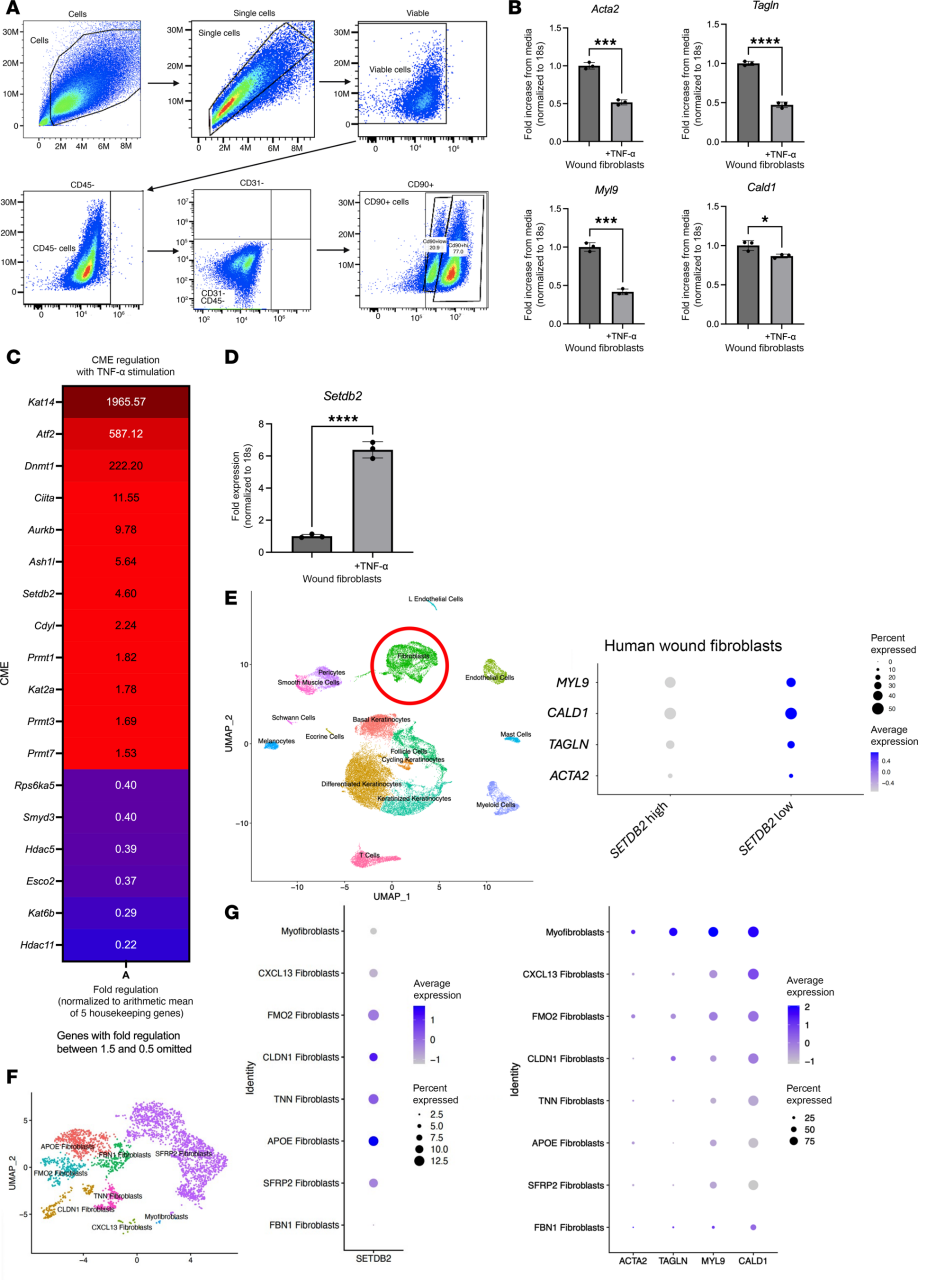
TNF-α represses myofibroblast gene expression and increases Setdb2 expression in wound fibroblasts. (**A**) Flow gating on wound fibroblasts that were isolated from wounds 7 days after injury in C57BL/6 mice utilizing magnetic separation for Tie2^–^, Ter119^–^, CD45^–^, CD31^–^, and EPCAM^–^ cells. Cells were plated and passaged 3 times prior to flow cytometry (*n* = 5 mice). (**B**) *Acta2*, *Tagln*, *Myl9*, and *Cald1* gene expression in wound fibroblasts isolated 7 days after wounding following TNF-α stimulation for 6 hours (25 ng/μL) (*n* = 4 mice/group, pooled and run in triplicate). (**C**) Unbiased epigenetic array comparing expression of chromatin modifying enzymes in dermal fibroblasts following 6 hours of TNF-α (25 ng/μL) stimulation with control. Chromatin modifying enzymes with less than 1.5- to 0.5-fold regulation were excluded. Fold regulation was normalized to arithmetic mean of 5 housekeeping genes (*n* = 4 mice/group, run in singlicate). (**D**) *Setdb2* expression in wound fibroblasts 7 days after wounding following TNF-α (25 ng/μL) stimulation for 6 hours (*n* = 4 mice/group, run in triplicate). (**E**) Cluster analysis uniform manifold approximation and projection (UMAP) of single-cell RNA sequencing from human T2D and non-T2D wounds showed 10 unique cell clusters (representative). Dot plots detailing *ACTA2*, *TAGLN*, *CALD1*, and *MYL9* gene expression between human wound fibroblasts expressing high amounts of *Setdb2* compared with those expressing low levels of *Setdb2* (*n* = 10). (**F**) Cluster analysis UMAP of single-cell RNA sequencing from human T2D and non-T2D wounds showed 8 unique fibroblast cell clusters (representative) (*n* = 10). (**G**) Dot plots detailing *SETDB2*, *ACTA2*, *TAGLN*, *CALD1*, and *MYL9* gene expression between human wound fibroblast subtypes (*n* = 10). *P < 0.05, ***P < 0.001, ****P < 0.0001. Data are presented as the mean ± SEM. Data were first analyzed for normal distribution, and if data passed the normality test, 2-tailed Student’s *t* test was used. Representative figures are displayed for panel **B** and **D**, which were repeated 3 times independently.

Fibroblast to myofibroblast transition is critical in normal tissue repair; however, the precise molecular mechanisms that control this transition in wounds are unclear. Cytokine stimulation has been found to regulate this transition, and in particular, TNF-α stimulation has been shown to induce (24) or repress myofibroblast gene expression (25), depending on the cell- and tissue-specific context (26–28). TNF-α is a highly pleiotropic cytokine present in wound tissue and is well documented to increase following injury. However, its role in fibroblast to myofibroblast transition and the downstream signaling pathway that controls this transition in wounds is not well defined. To examine the role of TNF-α on myofibroblast transition in our murine model of wound healing, we used a 6 mm punch biopsy wound model on C57BL/6 mice to induce an acute injury (29) and isolated fibroblasts via magnetic separation utilizing negative selection for Tie2, Ter119, CD45, CD31, and EPCAM on day 7 postinjury (Figure 1A) (30). Wound fibroblasts were passaged and then subjected to TNF-α stimulation (25 ng/μL) for 6 hours and were then examined for a panel of genes associated with myofibroblast transition including *Acta2*, as well as *Tagln*, *Cald1*, and *Myl9* (31). We found that TNF-α stimulation significantly repressed *Acta2*, *Tagln*, *Cald1*, and *Myl9* myofibroblast gene expression in wound fibroblasts (Figure 1B).

Next, we examined the molecular mechanisms by which myofibroblast genes were being repressed following TNF-α stimulation. Since our group and others have identified that CMEs can control cell plasticity in wound tissue (32–34), we examined whether epigenetic enzymes may be regulating myofibroblast gene expression, and hence, fibroblast to myofibroblast transition in response to TNF-α. In order to examine this, fibroblasts were isolated in vivo from dermis and were stimulated with TNF-α (25 ng/μL) for 6 hours, and a 96-well epigenetic superarray was performed (QIAGEN). We found that the histone methyltransferase *Setdb2* was among the highest CMEs upregulated following TNF-α stimulation (Figure 1C). To confirm the superarray finding, we stimulated isolated wound fibroblasts with TNF-α (25 ng/μL) for 6 hours, and identified, via quantitative PCR (qPCR), nearly a 6-fold upregulation of *Setdb2* (Figure 1D). SETDB2 has been shown to repress genes’ expression via trimethylation at the H3K9 site (H3K9me3) in various cells and tissues (35–37). Next, to determine the translational potential of these findings, we utilized scRNA-Seq of human wounds. Wound fibroblasts were identified as published previously, then stratified by high or low SETDB2 expression (22, 38). Wound fibroblasts that expressed low amounts of *Setdb2* had high expression of myofibroblast genes; fibroblasts with high levels of *Setdb2* demonstrated decreased expression of myofibroblast genes (Figure 1E). Additionally, fibroblasts were stratified by the other CMEs that were strongly upregulated in the unbiased epigenetic array (*Kat14*, *ATF2*, *Ciita*, *Aurkb*, *Dmnt1*, and *Ash1l*), and none had strong correlations with myofibroblast genes in fibroblasts in our human scRNA-Seq that were supportive of a direct mechanism with their known CME directionality (Supplemental Figure 1; supplemental material available online with this article; https://doi.org/10.1172/jci.insight.190836DS1). To determine the relationship between SETDB2 and myofibroblast gene expression in all fibroblast subtypes, 8 unique fibroblast subtypes were identified (Figure 1F). SETDB2 expression was low in myofibroblasts, which is consistent with its observed inverse regulation with myofibroblast genes. Setdb2 expression varied across the rest of the fibroblast subtypes, and, in general, subtypes that had high expression of *SETDB2* had low expression of myofibroblast genes (Figure 1G). Taken together these results suggest that TNF-α represses myofibroblast transition potentially via an upregulation of the CME, *Setdb2*.

### SETDB2-deficient fibroblasts express increased myofibroblast genes.

**Figure 2:**
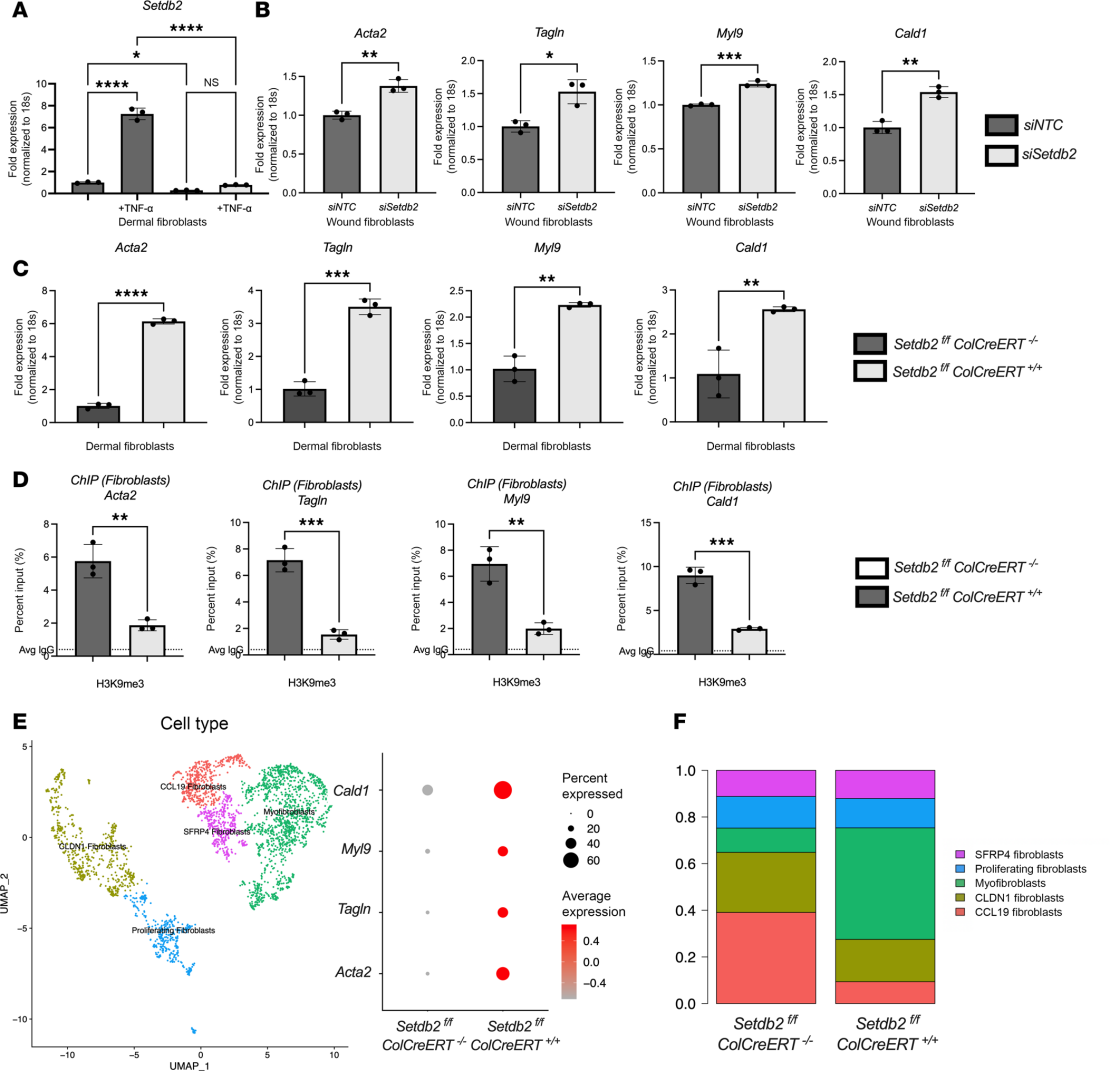
SETDB2-deficient fibroblasts express increased myofibroblast genes. (**A**) *Setdb2* gene expression in wound fibroblasts isolated from unwounded dermis of wild-type mice following 72 hours of siRNA transfection with nontargeting, negative control (siNTC) or siRNA specific to *Setdb2* (40 nM) (*n* = 5 mice/group, run in triplicate). (**B**) *Acta2*, *Tagln*, *Myl9*, and *Cald1* gene expression in wound fibroblasts isolated from day 7 of wild-type mice following 72 hours of siRNA transfection with siNTC or siRNA specific to *Setdb2* (40 nM) (*n* = 5 mice/group, run in triplicate). (**C**) *Acta2*, *Tagln*, *Myl9*, and *Cald1* gene expression in dermal fibroblasts isolated from *Setdb2^fl/fl^* *ColCreERT*^–*/*–^ and *Setdb2^fl/fl^* *ColCreERT^+/+^* (*n* = 5 mice/group, run in triplicate). (**D**) Real-time qPCR values for chromatin immunoprecipitation (ChIP) for H3K9me3 at the promoters of *Acta2*, *Tagln*, *Myl9*, and *Cald1* genes following 6 hours of TNF-α stimulation of *Setdb2^fl/fl^* *ColCreERT*^–*/*–^ and *Setdb2^fl/fl^* *ColCreERT^+/+^* dermal fibroblasts (*n* = 5 mice/group, run in triplicate). (**E**) Cluster analysis UMAP of single-cell RNA sequencing from *Setdb2^fl/fl^* *ColCreERT*^–*/*–^ and *Setdb2^fl/fl^* *ColCreERT^+/+^* day 7 wounds showed 5 unique fibroblast cell clusters (representative). Dot plots detailing *ACTA2*, *TAGLN*, *CALD1*, and *MYL9* gene expression between *Setdb2^fl/fl^* *ColCreERT*^–*/*–^ and *Setdb2^fl/fl^* *ColCreERT^+/+^* day 7 murine wound fibroblasts (*n* = 3 per group). (**F**) Histograms showing the proportion of fibroblasts by subtype in day 7 wounds isolated from *Setdb2^fl/fl^* *ColCreERT*^–*/*–^ and *Setdb2^fl/fl^* *ColCreERT^+/+^* (*n* = 3 per group). *P < 0.05, **P < 0.01, ***P < 0.001, ****P < 0.0001. Data are presented as the mean ± SEM. Data were first analyzed for normal distribution, and if data passed the normality test, 2-tailed Student’s *t* test was used. Panel **A** utilized Tukey’s multiple-comparison test, with a single pooled variance. Representative figures are displayed for panel **A**–**D**, which were repeated 3 times independently.

To further define the direct effect of SETDB2 on fibroblast to myofibroblast transition in wounds, we examined wound fibroblasts following siRNA knockdown of *Setdb2* (Dharmacon). Wound fibroblasts were isolated and treated with siRNA specific to *Setdb2* siRNA (siSetdb2) or scrambled nontargeting control RNA (siNTC) for 72 hours. Dermal fibroblasts treated with siSETDB2 demonstrated an 80% knockdown of *Setdb2* (Figure 2A). Wound fibroblasts isolated from wild-type mice treated with *siSetdb2* resulted in increased myofibroblast gene expression (*Acta2*, *Tagln*, *Cald1*, and *Myl9*) (Figure 2B).

Next, we examined this using a genetic, cell-specific model. We crossed our *Setdb2^fl/fl^* mice (36) with a Col1a2-CreER (ColCreERT) strain (39), thus producing mice with tamoxifen-induced, fibroblast-specific knockdown of SETDB2 (*Setdb2^fl/fl^* *ColCreERT^fl/fl^*). Following confirmation of decreased SETDB2 in fibroblasts after tamoxifen injections, dermal fibroblasts were isolated and examined for myofibroblast gene expression. Fibroblasts deficient in SETDB2 displayed higher levels of myofibroblast genes at baseline in vitro (Figure 2C). To examine the mechanism of SETDB2 repression of myofibroblast genes, we performed chromatin immunoprecipitation (ChIP) analysis of fibroblasts isolated from the dermis. SETDB2-deficient fibroblasts displayed less H3K9me3 at the promoters of myofibroblast genes (*Acta2*, *Tagln*, *Myl9*, and *Cald1*), suggesting that SETDB2 represses myofibroblast genes through H3K9me3 of the promoter sites of key myofibroblast genes (Figure 2D). Next, we harvested day 7 wounds from *Setdb2^fl/fl^* *ColCreERT^fl/fl^* and subjected them to single-cell sequencing. Fibroblasts from *Setdb2^fl/fl^* *ColCreERT^+/+^* mice expressed myofibroblast genes at higher levels than wild-type controls, confirming the findings in ex vivo cell culture (Figure 2E). Furthermore, 5 unique fibroblast cell cultures were identified, and the myofibroblast cell line was enriched in *Setdb2* *ColCreERT^+/+^* mice compared with wild-type control (Figure 2F). Taken together, these data suggest that SETDB2 represses classic myofibroblast gene expression via an H3K9-mediated mechanism in wound fibroblasts following tissue injury.

### Setdb2 expression is upregulated by TNF-α via JAK1,3/STAT3 signaling.

**Figure 3:**
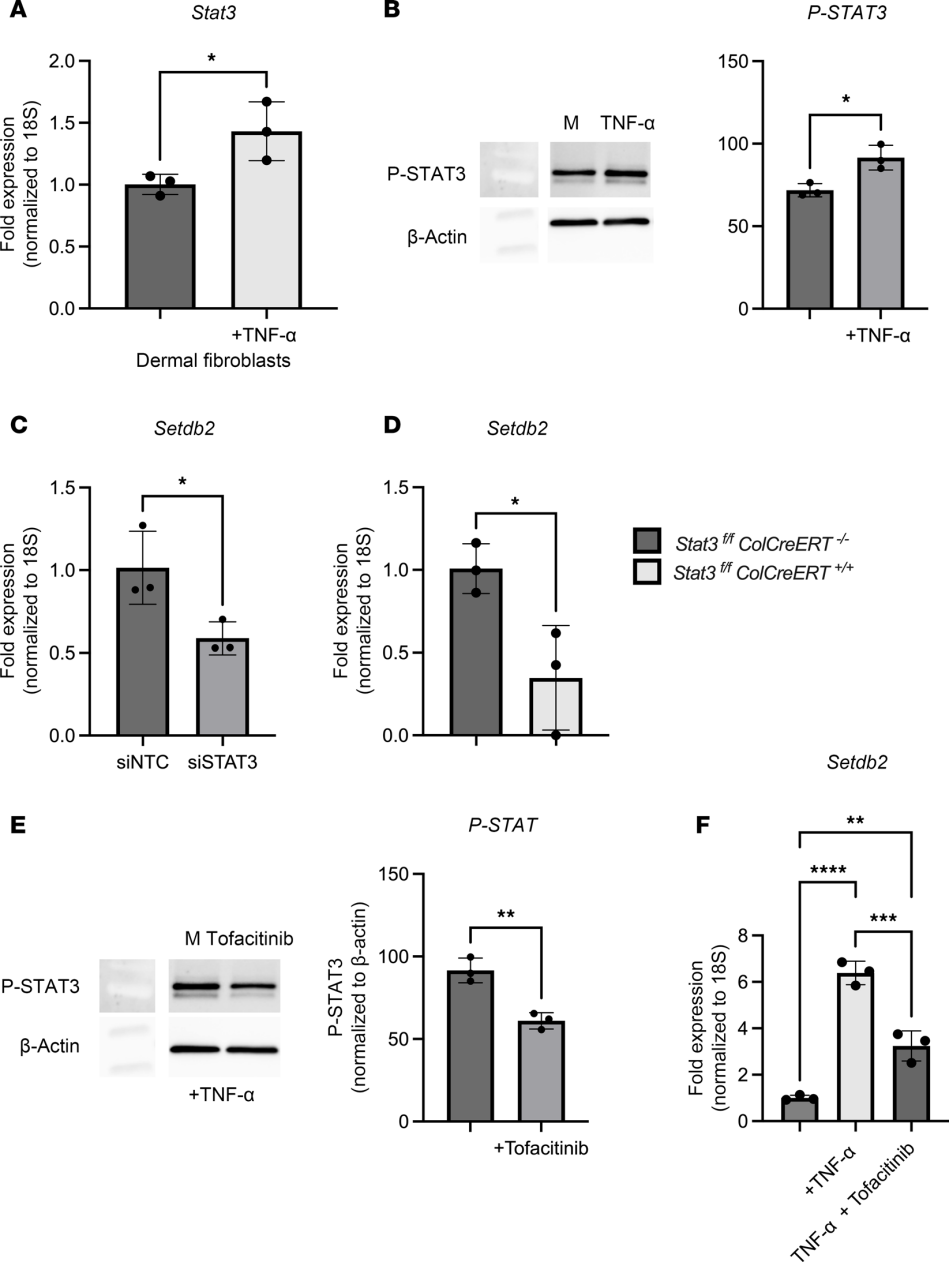
*Setdb2* expression is upregulated by TNF-α via JAK1,3/STAT3 signaling. (**A**) *Stat3* expression in dermal fibroblasts after 6 hours of TNF-α (25 ng/μL) stimulation, compared with control (*n* = 5 mice/group, run in triplicate). (**B**) Western blot for p-STAT3 and β-actin in isolated wound fibroblasts after 2 hours of stimulation of TNF-α (*n* = 5 mice/group, run in triplicate). (**C**) *Setdb2* gene expression in wound fibroblasts isolated from day 7 of wild-type mice following 72 hours of siRNA transfection with siNTC or siRNA specific to *Stat3* (siStat3) (40 nM) (*n* = 4 mice/group, run in triplicate). (**D**) *Setdb2* expression in isolated wound fibroblasts isolated from day 7 following injury from *Stat3^fl/fl^* *ColCreERT^+/+^* compared with *Stat3^fl/fl^* *ColCreERT*^–*/*–^ (*n* = 3 mice/group, run in triplicate). (**E**) Western blot for p-STAT3 and β-actin in isolated wound fibroblasts after 2 hours of stimulation of TNF-α or TNF-α+tofacitinib (50 nM) (*n* = 4 mice/group, run in triplicate). (**F**) *Setdb2* expression in isolated wound fibroblasts treated with TNF-α, or TNF-α+tofacitinib, compared with control (*n* = 4 mice/group, run in triplicate). *P < 0.05, **P < 0.01, ***P < 0.001, ****P < 0.0001. Data are presented as the mean ± SEM. Data were first analyzed for normal distribution, and if data passed the normality test, 2-tailed Student’s *t* test was used. Panel **F** utilized Tukey’s multiple-comparison test, with a single pooled variance. Representative figures are displayed for panels **A**, **C**, **D**, and **F**, which were repeated 3 times independently.

To examine the upstream signaling pathways that regulate *Setdb2* expression in wound fibroblasts following TNF-α stimulation, we investigated potential intermediate signal pathways known to be downstream from the TNF-α receptor. It is well established that the TNF-α receptor induces phosphorylation of a variety of STAT proteins in fibroblasts (40–42) and that *Setdb2* expression is regulated by STAT signaling in other cell types (36, 43, 44). Therefore, we isolated wound fibroblasts and stimulated them with TNF-α for 6 hours and examined *Stat(1–4)* transcription. The most upregulated *Stat* gene following TNF-α stimulation was *Stat3* (Figure 3A), and we found increased levels of active p-STAT3 in fibroblasts following TNF-α stimulation (25 ng/μL for 2 hours) (Figure 3B); there was minimal to no upregulation of *Stat1*, *Stat2*, or *Stat4* following TNF-α (25 ng/μL) stimulation for 6 hours (Supplemental Figure 2). Next, to determine the effects of STAT3 on *Setdb2* expression independent of STAT1, 2, and 4, we utilized siRNA knockdown of *Stat3* (siStat3) (Dharmacon) in isolated wound fibroblasts. Wound fibroblasts were treated with siStat3 or siNTC for 72 hours, and fibroblasts treated with siStat3 demonstrated reduced *Setdb2* expression compared with controls (Figure 3C). To confirm these results, we developed a genetic, fibroblast-specific knockdown of *Stat3* (*Stat3^fl/fl^ ColCreERT^fl/fl^*) and found that wound fibroblasts isolated on day 7 following injury from these mice expressed decreased *Setdb2* compared with littermate controls (Figure 3D). To examine JAK signaling upstream of STAT3, we utilized an FDA-approved small molecule inhibitor of Jak 1,3, tofacitinib (45). Wound fibroblasts isolated and treated with tofacitinib (50 nM) and stimulated with TNF-α for 2 hours displayed decreased conversion of STAT3 to p-STAT3 (Figure 3E). Furthermore, the treatment of dermal fibroblasts with tofacitinib also reduced TNF-α–induced upregulation of *Setdb2* (Figure 3F). These data suggest that TNF-α upregulates *Setdb2* through a JAK1,3/STAT3 mechanism and that inhibition of the JAK1,3/STAT3 pathway may reduce the upregulation of *Setdb2* in wound fibroblasts.

### Pro-inflammatory macrophages drive Setdb2 expression in fibroblasts.

**Figure 4:**
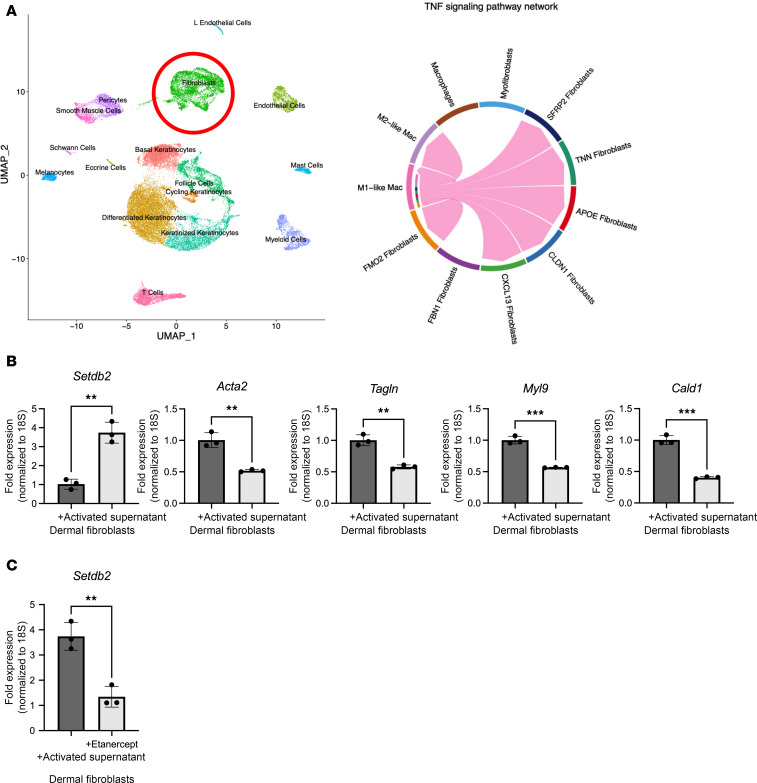
Pro-inflammatory macrophages drive *Setdb2* expression in fibroblasts. (**A**) Cluster analysis UMAP of single-cell RNA sequencing from human T2D and non-T2D wounds showed 10 unique cell clusters (representative), with both fibroblasts and macrophages identified. River plots depicting receptor-ligand interactions affect outgoing (signal source) TNF-α interactions from macrophages and incoming (signal responder) patterns from fibroblast subtypes. The thickness of the flow indicates the contribution of the cell group or signaling pathway to each latent pattern (*n* = 10). (**B**) *Setdb2*, *Acta2*, *Tagln*, *Myl9*, and *Cald1* expression in isolated dermal fibroblasts treated with supernatants from activated BMDMs or control BMDMs. Supernatants were mixed 1:1 with serum-free DMEM and applied to fibroblasts for 4 hours (*n* = 4 mice/group for fibroblasts, *n* = 8 mice/group for BMDMs, run in triplicate). (**C**) *Setdb2* expression in isolated dermal fibroblasts treated with supernatants from activated BMDMs or control BMDMs. Supernatants were mixed 1:1 with serum-free DMEM ± etanercept (4 μM) and applied to fibroblasts for 4 hours (*n* = 4 mice/group for fibroblasts, *n* = 8 mice/group for BMDMs, run in triplicate). **P < 0.01, ***P < 0.001. Data are presented as the mean ± SEM. Data were first analyzed for normal distribution, and if data passed the normality test, 2-tailed Student’s *t* test was used. Representative figures are displayed for panel **B** and **C**, which were repeated 3 times independently.

It is known that in chronic inflammatory wounds (i.e., T2D) fibroblast functions are impaired. Although it is known that the pro-inflammatory environment seen in T2D wounds is driven by alterations in immune cells (increased pro-inflammatory macrophages) (46, 47), the degree to which pro-inflammatory macrophages affect fibroblast function is unknown. Analysis of our human scRNA-Seq dataset demonstrated an absence of crosstalk between pro-inflammatory macrophages and myofibroblasts in T2D wounds, suggesting that the pro-inflammatory environment may repress fibroblast to myofibroblast transition (Figure 4A). To determine if pro-inflammatory macrophages are a primary regulator of *Setdb2* in wound fibroblasts, we first isolated bone marrow–derived macrophages (BMDMs) from C57BL/6 mice as we have previously described (48). Next, we polarized these BMDMs toward a pro-inflammatory phenotype using LPS (100 ng/mL) stimulation for 4 hours (49). Dermal fibroblasts were then incubated with supernatants from the pro-inflammatory BMDMs for 4 hours, and fibroblasts were then examined for *Setdb2* and myofibroblast gene expression. Fibroblasts treated with pro-inflammatory, activated BMDM supernatant demonstrated increased *Setdb2* expression and reduced myofibroblast gene expression, as compared with fibroblasts treated with unstimulated BMDM supernatant (Figure 4B). Addition of etanercept (4 μM, MedChemExpress), a TNF-α decoy receptor that decreases TNF-α receptor signaling to the activated BMDM supernatants for 4 hours prevented the increase in expression of *Setdb2*, suggesting that *Setdb2* is regulated by TNF-α in the inflammatory macrophage supernatants (Figure 4C). This suggests that pro-inflammatory macrophages may regulate *Setdb2* expression in wound fibroblasts, primarily through TNF-α signaling.

### TNF-α signaling is increased in diabetic wounds and drives Setdb2 expression in fibroblasts.

**Figure 5:**
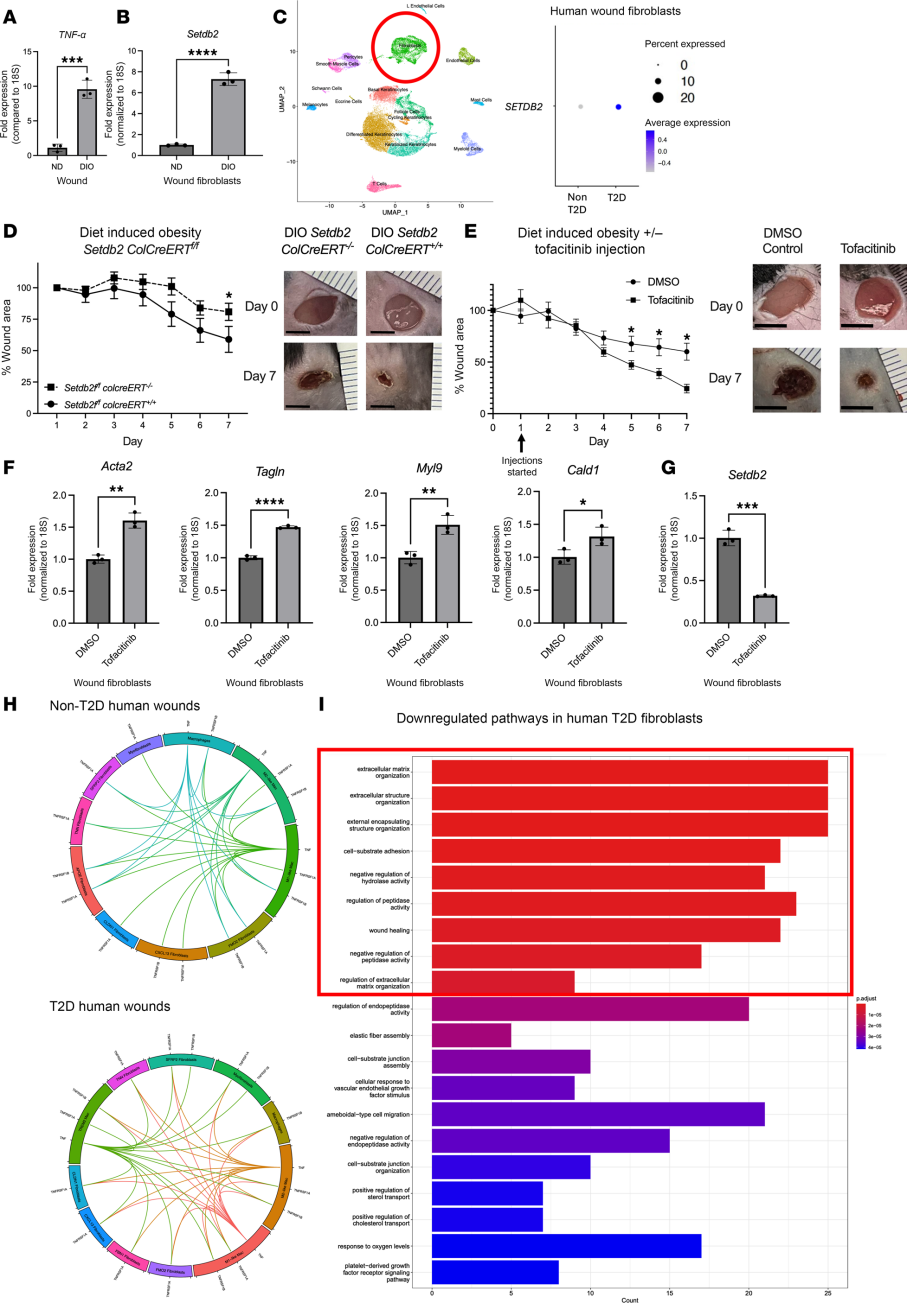
TNF-α signaling is higher in diabetic wounds, driving *Setdb2* expression. (**A**) Wound expression of *TNF-α* harvested on day 7 following tissue injury (DIO and littermate controls) (*n* = 4 mice/group, run in triplicate). (**B**) *Setdb2* *expression* in fibroblasts isolated day 7 following tissue injury (DIO and littermate controls) (*n* = 4 mice/group, run in triplicate). (**C**) Cluster analysis UMAP of single-cell RNA-Seq from human T2D and non-T2D wounds. Dot plot comparing *Setdb2* expression in human T2D with non-T2D wound fibroblasts (*n* = 10). (**D**) Wound healing curve for DIO *Setdb2^fl/fl^* *ColCreERT^fl/fl^* mice. Mice were wounded via 6 mm punch biopsy and images were taken daily. Scale bar is 4 mm (*n* = 10/group for *Setdb2^fl/fl^* *ColCreERT*^–*/*–^ and 5 for *Setdb2^fl/fl^* *ColCreER^+/+^*). (**E**) Wound healing curve for DIO mice injected daily with tofacitinib (1 mg/kg) or DMSO control. Tofacitinib injections were started on day 1. Scale bar is 4 mm (*n* = 6/group). (**F**) *Acta2*, *Tagln*, *Myl9*, and *Cald1* gene expression from wound fibroblasts isolated from mice injected daily with tofacitinib or DMSO control following wounding (*n* = 5 mice/group, run in triplicate). (**G**) *Setdb2* gene expression from wound fibroblasts isolated from mice injected daily with tofacitinib or DMSO control following wounding, following stimulation with TNF-α for 6 hours (*n* = 5 mice/group, run in triplicate). (**H**) Receptor-ligand plots depicting TNF-α receptor-ligand interactions in human scRNA-Seq between wound macrophage and wound fibroblast subtypes (*n* = 10). (**I**) Significantly downregulated pathways in human T2D wound fibroblasts compared with non-T2D wound fibroblasts. Red box indicates most strongly downregulated pathways (*n* = 10). *P < 0.05, **P < 0.01, ***P < 0.001, ****P < 0.0001. Data are presented as the mean ± SEM. Data were first analyzed for normal distribution, and if data passed the normality test, 2-tailed Student’s *t* test was used. Representative figures are displayed for panel **A**, **B**, **F**, and **G**, which were repeated 3 times independently.

To translate our findings to T2D wound repair, where there is decreased fibroblast to myofibroblast transition, we used a physiologic murine model (diet-induced obesity, DIO) similar to T2D where male mice are fed a high-fat diet (HFD) for 12 weeks and develop glucose intolerance and insulin resistance (48). *Tnf-α* expression was higher in wounds harvested 7 days after injury from DIO mice, as compared with controls, similar to what our group and others have found in human and murine T2D tissue (Figure 5A) (19, 50). Furthermore, when we examined wound fibroblasts isolated from DIO mice on day 7 postinjury, we found that *Setdb2* expression was upregulated in DIO wound fibroblasts compared with control (Figure 5B). Next, we examined our human scRNA-Seq dataset and found that human wound fibroblasts from T2D wounds displayed increased expression of *Setdb2* compared with fibroblasts from non-T2D wounds (Figure 5C). Next, in order to determine if reduction of SETDB2 in wound fibroblasts can improve DIO wound repair, *Setdb2^fl/fl^* *ColCreERT^fl/fl^* mice were started on a DIO diet and were subjected to 6 mm punch biopsy wounding. Wound healing was monitored using ImageJ (National Institutes of Health) software as previously described (48). DIO mice with fibroblasts deficient in *Setdb2* healed significantly better than littermate controls (Figure 5D). Since there is no known direct pharmacologic inhibitor of SETDB2, mice were injected with tofacitinib (1 mg/kg) daily starting on day 1 in order to determine if the pharmacologic repression of the TNF-α/STAT3/SETDB2 axis had translational relevance for wound healing. Mice injected with tofacitinib demonstrated improved wound healing compared with those injected with DMSO control (Figure 5E), and fibroblasts isolated from the wounds of mice injected daily with tofacitinib displayed increased myofibroblast genes compared with control (Figure 5F). Furthermore, wound fibroblasts from mice injected with tofacitinib expressed decreased *Setdb2* in the presence of ex vivo stimulation with TNF-α (25 ng/μL) for 6 hours (Figure 5G). Analysis of receptor-ligand interactions in human wounds showed an increase in TNF-α signaling among fibroblasts in T2D wounds (41 interactions), compared with non-T2D (32 interactions) (Figure 5H). Gene ontology pathway analysis comparing human fibroblasts from T2D wounds with non-T2D wounds demonstrated several had many significantly downregulated pathways associated with myofibroblast function, including extracellular matrix structure/organization, cell substrate adhesion, elastic fiber assembly, and wound healing, among others (Figure 5I). These data suggest *Setdb2* is increased in T2D wound fibroblasts, leading to reduced fibroblast to myofibroblast transition in T2D wounds, and inhibition of the TNF-α/STAT3/SETDB2 pathway may improve fibroblast function in diabetic wound repair.

## Discussion

In this study we define a role for SETDB2 in fibroblasts, as a regulator of fibroblast to myofibroblast transition in wounds. Specifically, we show that TNF-α upregulates *Setdb2* in wound fibroblasts through JAK1,3/STAT3 and SETDB2 functions to suppress transcription of key genes necessary for fibroblast to myofibroblast transition, including *Acta2*, *Tagln*, *Myl9*, and *Cald1*. We show that fibroblasts treated with supernatants from pro-inflammatory macrophages, similar to T2D wounds, increase *Setdb2* and repress myofibroblast genes and that treatment with a commercially available TNF-α decoy receptor can reduce upregulation of *Setdb2*. Finally, we show that the TNF-α/SETDB2 pathway is upregulated in T2D wound fibroblasts and that fibroblast-specific knockdown of *Setdb2* or pharmacologic inhibition of JAK1,3/STAT3 improves wound healing in diabetic mice and leads to increased myofibroblast gene expression in T2D wounds. This suggests a TNF-α/SETDB2 cell-specific therapy may improve T2D wound healing.

Our group has previously investigated the pathophysiology associated with epigenetic alterations in T2D wounds that result in pathologic cell function. The focus of the majority of our and others’ work has been on immune cells and has unveiled a myriad of CMEs that regulate cell plasticity (19, 21, 34, 51). However, the role of CMEs in structural cells in wounds is less well studied. Although *Setdb2* has been examined in other cell types, this is the first investigation in fibroblasts (17–20). In this study, we show that *Setdb2* in fibroblasts directly regulates classic myofibroblast gene expression, including *Acta2*, *Tagln*, *Myl9*, and *Cald1*. Myofibroblasts function to produce extracellular matrix proteins and contract wounds, specifically through *Acta2*, which incorporates into stress fibers to pull wound edges together (52, 53). Myofibroblasts, specifically fibroblasts that express high amounts of *Acta2* and its gene product α-SMA, are highly correlated with wound healing (54, 55), and reduced myofibroblasts are a hallmark of chronic wounds, such as in T2D. Despite the clear relationship between *Acta2* gene expression, fibroblast to myofibroblast differentiation, and wound healing, there have been limited prior investigations into the potential epigenetic regulation of the myofibroblast gene set (15). In this regard, this study explores an unknown area by examining molecular mechanisms of the reduction in the number and function of myofibroblasts in diabetic wounds.

Here, we show that TNF-α increases *Setdb2* in wound fibroblasts through JAK1,3/STAT3 signaling. This was not unexpected since TNF-α receptor stimulation induces phosphorylation of a variety of STAT proteins in fibroblasts (40–42), and *Setdb2* expression is regulated by STAT signaling in other cell types (36, 43, 44). Since *Setdb2* does not have any direct pharmacologic inhibitors, the identification of JAK1,3/STAT3 as an upstream pathway increases the translational relevance of this work and allows for future studies targeting JAK1,3/STAT3 to alter fibroblast phenotype.

Fibroblasts are a notoriously heterogeneous cell population and are not unified by any cell surface marker. While there are many populations of fibroblasts in wounds, investigations aimed at understanding the epigenetic alterations in fibroblasts have been hampered by the lack of clear, well-defined cellular pathways in fibroblasts during wound healing (56). This investigation focused on the epigenetic regulation of the most well-defined cellular pathway for fibroblasts in wound healing, fibroblast to myofibroblast transition. We show that knockdown of SETDB2 leads to increased myofibroblast gene expression in all fibroblast populations in vitro and that in vivo knockdown of SETDB2 leads to an increase in myofibroblasts as identified by scRNA-Seq. While we do show that SETDB2 has an inverse correlation with myofibroblast gene expression across all fibroblast subtypes, the relevance of the TNF-α/STAT3/SETDB2 axis on cellular phenotype in each individual fibroblast subtype remains unclear. Future studies with targeted in vivo and in vitro analyses of fibroblast function are needed to define clear roles for the main fibroblast subtypes in the dermis; these studies may be informed from readily available single-cell multiomic datasets that provide high-quality, granular data on dermal cell types (6, 57, 58).

In T2D, alterations in circulating immune cells prior to injury (59, 60), as well as higher levels of glucose, advanced glycolytic end products, and bacterial counts (23), lead to a drastically different wound environment in wounds. It has been established by our group and others that many factors in T2D polarize both resident and recruited macrophages to a pro-inflammatory subtype, which overproduce TNF-α in T2D wounds (61). Little is known about how these pro-inflammatory immune cells influence structural cells in T2D wounds. Using BMDM conditioned media we show that the pro-inflammatory environment of macrophages (similar to that seen in T2D wounds) represses myofibroblast gene expression and increase *Setdb2* expression, which was reversible with a commercially available inhibitor of TNF-α. These findings are corroborated in our human scRNA-Seq showing higher levels of TNF-α receptor-ligand interactions between macrophages and fibroblasts in human T2D wounds compared with non-T2D wounds and an absence of pro-inflammatory macrophage crosstalk with myofibroblasts in T2D wounds. Our work on macrophage-fibroblast interaction builds on the existing literature by identifying how known, pathologic inflammation impairs normal structural cell function in wound healing.

Although this work provides insight into the TNF-α/SETDB2 axis that regulates fibroblast to myofibroblast transition in wounds, there are a few limitations that should be addressed. First, we chose to focus on 4 genes that are well established as indicators of myofibroblasts, including the most classic gene for fibroblast to myofibroblast transition, *Acta2* (31). There may be additional genes that are important for fibroblast to myofibroblast transition that are not regulated by *Setdb2*. Furthermore, we chose to study wound fibroblasts isolated from day 7 after wounding, which was informed by the literature, where this time is firmly in the proliferative phase of wound healing in murine models, which should be optimal to study myofibroblast gene expression and transition (54, 62, 63). While day 7 is an appropriate time point based off of prior literature, it is possible that the TNF-α/SETDB2 axis is not relevant at all time points following injury. While we show that the TNF-α/STAT3/SETDB2 axis regulates myofibroblast genes across all wound fibroblasts, the relevance of this pathway in each individual fibroblast subtype is not confirmed. Furthermore, fibroblasts are extremely heterogeneous between different tissues, and it remains unclear if the TNF-α/STAT3/SETDB2 axis is relevant in myofibroblast gene expression in other tissues. Finally, while we do show that SETDB2 directly trimethylates the promoters of myofibroblast genes to repress gene expression, we cannot rule out additional, indirect pathways that regulate SETDB2 or its interactions with other CMEs or transcription factors that may influence myofibroblast gene expression.

**Figure 6:**
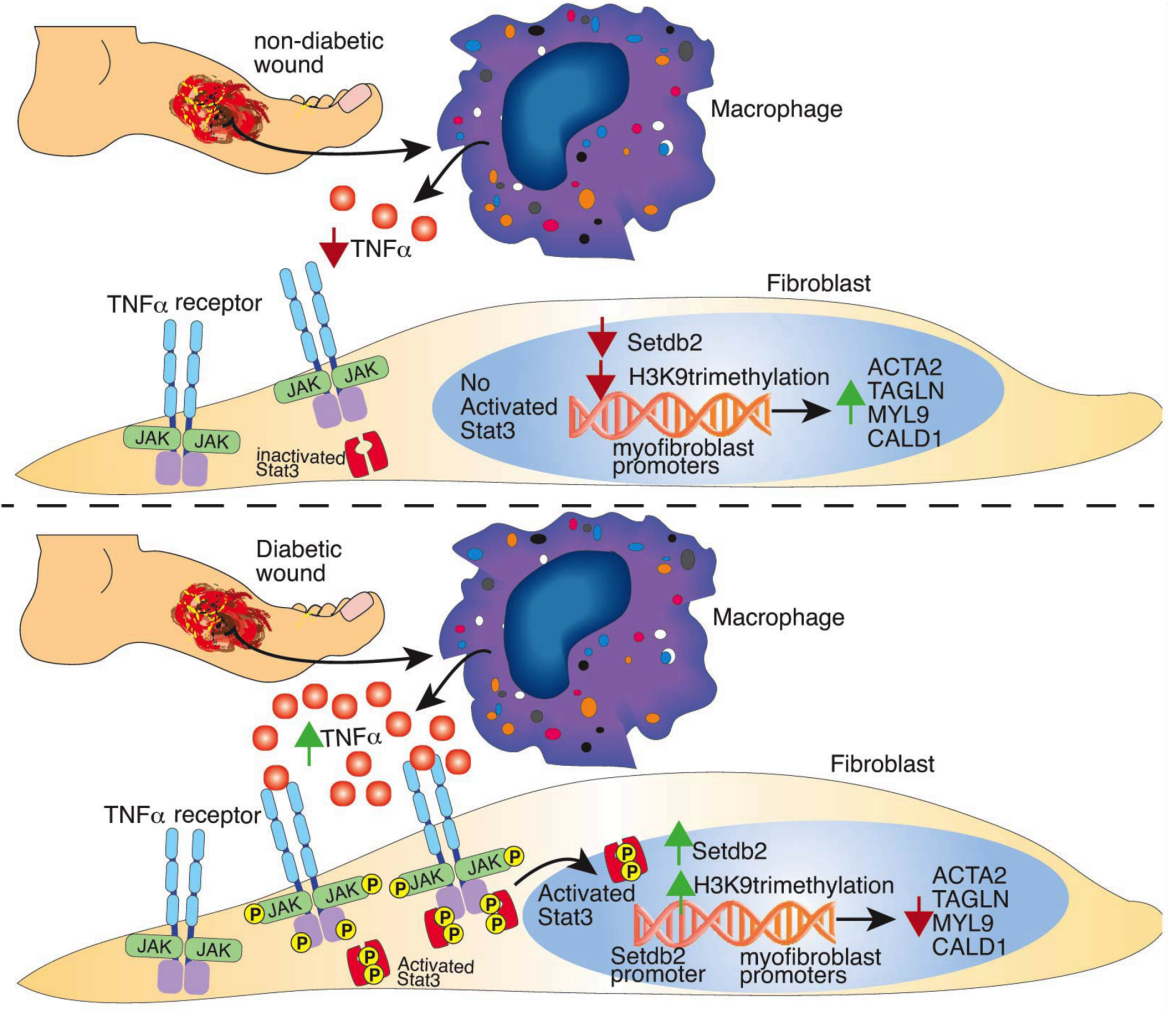
Study schematic.

In summary, we show that TNF-α signaling in fibroblasts increases *Setdb2*, which functions to repress fibroblast to myofibroblast transition. *Setdb2* directly trimethylates H3K9 at the promoter sites of myofibroblast genes and reduces gene expression. We find that JAK1,3/STAT3 signaling is a key intermediate in the TNF-α/Setdb2 pathway, further advancing the translational application of this work. We show that the TNF-α/STAT3/SETDB2 pathway is upregulated in T2D wound fibroblasts and that fibroblast-specific knockdown of SETDB2 or pharmacologic inhibition of JAK1,3/STAT3 signaling can improve T2D wound healing (Figure 6). Further investigation into a cell-specific therapeutic target to improve wound healing in patients with T2D would be both relevant and timely.

## Methods

### Sex as a biological variable.

Sex was not considered as a biological variable in all reported human data. Only males were used in the murine studies, since female C57BL/6 mice do not exhibit the DIO phenotype when placed on an HFD.

### Mice.

All mice were maintained at the University of Michigan in the Unit for Laboratory and Animal Medicine (ULAM). Mouse experiments were conducted with approval from our IACUC, and all regulatory and safety standards were strictly adhered to. C57BL/6 mice were obtained at 6–7 weeks of age from Jackson Laboratory and maintained in breeding pairs at the ULAM facilities. Mice with the flox sites in the *Setdb2* (64) or *Stat3* (Jackson Laboratory strain 16923) gene (*Setdb2^fl/fl^* *ColCreERT*; *Stat3^fl/fl^* *ColCreERT*) were generated by mating *gene^fl/fl^* mice with *ColCreERT* mice (Jackson Laboratory strain 029567) (21, 39, 65). Animals were housed in a barrier facility on a 14-hour light/10-hour dark cycle (ambient temperature of 22°C) with free access to water, food (Lab Supply Lab Diet Rodent 5001), and bedding (Andersons Lab Bedding Bed-o’Cobs combo).

### Murine model of diabetes.

To induce a “prediabetic” state, male C57BL/6 mice were maintained on a standard HFD (60% kcal saturated fat, 20% protein, 20% carbohydrate, Research Diets, Inc.) for 12–18 weeks to induce the DIO model of T2D as previously described (48). After the appropriate period, HFD-fed (DIO) mice developed obesity and insulin resistance with fasting blood sugars in the mid-200s and elevated insulin levels (48). All animals underwent procedures at 20–32 weeks of age with IACUC approval. For these experiments only male mice were used because female mice do not develop DIO. Number of mice used per experiment can be found in the figure legend of each corresponding experiment.

### Murine wound healing model.

Mice were anesthetized, dorsal hair was removed with Veet (Reckitt Benckiser) and rinsed with sterile water, and 2 full-thickness back wounds were created by 6 mm punch biopsy without wound splinting.

### MACS of murine wound fibroblasts.

Briefly, after harvest of wound tissue from mice, the wounds were digested in 1% trypsin overnight at 4°C. The following day, tissue was morcellated, and wounds were digested in a suspension of 3.5% collagenase IV (Worthington) at 37°C for 30 minutes. DMEM (Corning, catalog10-017-CV) + 10% FBS (Atlas Biologicals, catalog FP-0500-A) was added to stop the digestion, and wounds were passed through a 100 μm filter (Corning, catalog 431752). The single-cell solution was subjected to negative selection for Tie2, Ter119, CD45, CD31, and EPCAM (30).

### Ex vivo cell culture of murine wound fibroblasts.

The negative fraction of the MACS reaction above was resuspended in DMEM + 10% FBS + penicillin/streptomycin (Gibco, catalog 15140-122) + amphotericin (Gibco, catalog 15290-018) + primocin (InvivoGen, catalog Ant-pm-1) and plated into a T25 dish and cultured at 37°C for at least 3 days. Subsequently, the cells were lifted with 0.25% trypsin (Gibco, 1505-057) and replated as necessary. Media were changed every third day, and cells were passaged every 3–5 days depending on confluence. Cells were passaged 1–10 times prior to use in experiments. Prior to in vitro experimentation, cells were serum starved with DMEM + 0.1% BSA (Sigma-Aldrich, catalog A9418-100g) + penicillin/streptomycin overnight. Cells were stimulated with TNF-α at 25 ng/μL (R&D Systems, catalog 410MT), tofacitinib at 50 nM (Cayman Chemical, catalog 11598), or sham in serum-free media for 4, 6, or 8 hours the following day.

### BMDM culture.

Bones of unwounded mice were flushed with RPMI (Corning, catalog 15-040-CV) supplemented with 10% FBS/penicillin/streptomycin. Tissue was incubated in 0.16% collagenase (Worthington) at 37°C. Cells underwent red blood cell lysis followed by Ficoll separation. Leukocytes were harvested from the buffy coat. Macrophages were cultured from human BM in the presence of 25 ng/mL M-CSF, 2.5 ng/mL GM-CSF, 50 ng/mL SCF, and 20 ng/mL IL-3 (R&D Systems) as described previously (66). BMDMs were activated to a pro-inflammatory subtype by stimulation with LPS at 100 ng/L for 4 hours or sham. After 4 hours, media were decanted and then washed once with PBS, and new RPMI +10% FBS and penicillin/streptomycin were added to the wells for an additional 24 hours to condition media. Fibroblasts were subsequently stimulated with the conditioned media mixed with serum-free DMEM for 4 hours ± etanercept at 4 μM (MedChemExpress, catalog HY-108847).

### Flow cytometry.

Wound cells were first lifted from T150 flasks following 1–10 passages using 0.25% trypsin (Gibco, 1505-057). Cells were first stained with a fixable viability stain (BD Horizon viability stain 510; 564406; 1:1,000 dilution) and then washed 2 times with cold PBS. Cells were then resuspended in flow buffer (500 mL PBS, 2% FBS, 1 mM EDTA), and Fc receptors were blocked with anti-CD16/32 (InVivoMAb, catalog BE0307, 1:200 dilution) prior to surface staining. Monoclonal antibodies used for surface staining included PE CD90.2 (BioLegend 140308), PECy7 CD45 (BioLegend 109829), APC CD31 (BioLegend 102509), and PerCpCy5.5 CD326 (BioLegend 118220). Following surface staining, cells were washed twice. Next, cells were fixed in 1% formalin for flow cytometry. Samples were then acquired on a 4-Laser Novocyte Flow Cytometer (Agilent). Data were analyzed using FlowJo software version 10.0 (FlowJo LLC) and compiled using Prism software 9.2 (GraphPad). To verify gating and purity, all populations were routinely backgated.

### RNA isolation.

Total RNA extraction was performed with TRIzol (Invitrogen, Thermo Fisher Scientific) using the manufacturer’s directions. RNA was extracted using chloroform, isopropanol, and ethanol. Superscript IV Reverse Transcriptase (Thermo Fisher Scientific) kits were used to synthesize cDNA from extracted RNA. We used cDNA primers for *Setdb2* (*Mm01318752_m1*), *Stat1* (*Mm00803077_m1*), *Stat2* (*Mm00490880_m1*), *Stat3* (*Mm01219775_m1*), *Stat4* (*Mm00448890_m1*), *Acta2* (*Mm00490880_m1*), *Tagln* (*Mm00441661_g1*), *Myl9* (*Mm01251442_m1*), *Cald1* (*Mm01129541_m1*), *Tnf* (*Mm00443258_m1*), and 18s as the internal control. Data were analyzed relative to 18s ribosomal RNA (2^-ΔCt^). All samples were assayed in technical triplicate. The threshold cycle values were used to plot a standard curve. Data are representative of 2 to 3 independent experiments and were compiled in Microsoft Excel and presented using Prism software (GraphPad).

### ChIP assay.

ChIP assay was performed as described previously (48). Briefly, cells were fixed in 1% paraformaldehyde and lysed and sonicated using a probe sonicator (Branson) to generate 300–500 bp fragments. Samples were then incubated overnight in anti-H3K9me3 antibody (ab8850, Abcam) or isotype control (rabbit polyclonal IgG ab171870, Abcam) in parallel followed by addition of protein A–Sepharose beads (Thermo Fisher Scientific). Beads were washed and bound; DNA was eluted and purified using phenol/chloroform/isoamyl alcohol extraction followed by ethanol precipitation. H3K9me3 deposition was measured by qPCR using 2× SYBR PCR mix (Invitrogen, Thermo Fisher Scientific) and primers targeting the promoter sites in the *Acta2*, *Tagln*, *Cald1*, and *Myl9* promoters. Primers were designed using the Ensembl genome browser to search the *Acta2*, *Tagln*, *Cald1*, and *Myl9* promoters for within the promoter region, and then National Center for Biotechnology Information (NCBI) Primer-BLAST was used to design primers that flank this site. The following primers were used to amplify DNA in samples: Acta2: 5′-TCCTGTTTCGGGAGCAGAAC-3′ and 5′-AGCTGGCCGTTCACTCTAAC-3′. Tagln: 5′-GGATGACCCATGTTCTGCCA-3′ and 5′-GTTTGGCCCGACAAAGGAAC-3′. Myl9: 5′-CTGCCTCTACCGAGGAGATG-3′ and 5′-ACTGAGCCCTTCAGTGCTTG-3′. Cald1: 5′-ATCCTGAGCATGCCTAGGGA-3′ and 5′-TAAAACTGCAGACCGCCCTT-3′.

### Bulk RNA sequencing and scRNA-Seq analyses.

Generation of single-cell suspensions for scRNA-Seq was performed in the following manner: Following informed consent from patients, skin was harvested via punch biopsy from diabetic and nondiabetic control wounds. Samples were incubated overnight in 0.4% Dispase (Life Technologies, Thermo Fisher Scientific) in HBSS (Gibco, Thermo Fisher Scientific) at 4°C. The epidermal and dermal layers were separated. The epidermis was digested in 0.25% Trypsin-EDTA (Gibco, Thermo Fisher Scientific) with 10 units/mL DNase I (Thermo Fisher Scientific) for 1 hour at 37°C and subsequently quenched with FBS (Atlanta Biologicals) and strained through a 100 μM mesh strainer (Corning, catalog 431752). The dermis was minced, digested in 0.2% Collagenase II (Life Technologies, Thermo Fisher Scientific), and 0.2% Collagenase V (Millipore Sigma) in plain RPMI medium for 1.5 hours at 37°C, then strained through a 100 μM mesh. Epidermal and dermal cells were combined in a 1:1 ratio for scRNA-Seq by the University of Michigan Advanced Genomics Core on the 10x Genomics Chromium System. Libraries were sequenced on the Illumina NovaSeq 6000 sequencer. NovaSeq was used as the sequencing platform to generate 151 bp paired-end reads. We conducted adapter trimming and quality control procedures as described previously (67). The reads were then mapped using STAR (68) to build human GRCh37, and gene expression levels were quantified and normalized by HTSeq (69) and DESeq2 (70). Negative binomial models in DESeq2 were used to conduct differential expression analysis. For scRNA-Seq data accession, the numbers include GSE154556 and GSE154557 (NCBI Gene Expression Omnibus [GEO]). ScRNA-Seq data processing, including quality control, read alignment, and gene quantification, was conducted using 10x Genomics Cell Ranger software. Seurat was then used for normalization, data integration, and clustering analysis. All clustered cells were mapped to corresponding cell types by matching cell cluster gene signatures with putative cell type–specific markers.

### PCR arrays.

The murine CME array was purchased from QIAGEN (catalog PAMM-085Z). RNA was DNase-digested using the RNeasy Mini Kit and reverse transcription RT2 first strand kit (QIAGEN, catalog 330421). Reverse transcription PCR was performed according to the manufacturer’s instructions, and gene expression was normalized to the arithmetic mean of multiple housekeeping genes.

### Statistics.

GraphPad Prism software (RRID:SCR_002798) version 9.2 was used to analyze the data. For all single-group comparisons, if data passed the normality test, we used a 2-tailed Student’s *t* test. Otherwise, data were analyzed using the Mann-Whitney *U* test. Experiments involving multiple comparisons utilized Tukey’s multiple-comparison test, with a single pooled variance. All data are representative of at least 3 independent experiments as detailed in the figure legends. A *P* value of less than or equal to 0.05 was significant.

### Data availability.

The human scRNA-Seq data are publicly available, and the ascension numbers include GSE154556 and GSE154557 (NCBI GEO). Graphically represented data in this manuscript are available in the Supporting Data Values supplemental file.

### Study approval.

Human single-cell sequencing of diabetic and nondiabetic wounds was conducted under University of Michigan IRB Study HUM00098915. All mice used were on a C57BL/6 background. Mice were housed at the University of Michigan Biomedical Sciences and Research Building in the ULAM, a pathogen-free animal facility. Mouse experiments were conducted with approval from the University of Michigan IACUC (protocol no. PRO00009811), and all regulatory and safety standards were strictly adhered to.

## Author contributions

TMB, KAG, KDM, SDB, JS, JEG, JYM, and FMD designed the experiments. TMB, KDM, JS, ADJ, JYM, RB, LCT, and COA performed experiments. TMB, RB, KDM, COA, ADJ, JYM, LCT, and TMB analyzed data. TMB and KAG wrote the manuscript. TMB, KDM, SDB, JS, ADJ, COA, JYM, LDH, RB, LCT, QL, HZ, SK, JEG, FMD, and KAG reviewed, edited, and accepted the manuscript in its final form.

## Funding support

This work is the result of NIH funding, in whole or in part, and is subject to the NIH Public Access Policy. Through acceptance of this federal funding, the NIH has been given a right to make the work publicly available in PubMed Central.

- National Institutes of Health grant R01-HL137919 (KAG).
- National Institutes of Health grant R01-DK124290 (KAG).
- National Institutes of Health grant R01-AR079863 01 (KAG).
- National Institutes of Health grant R01-HL156274-01A1 (KAG).
- National Institutes of Health grant R01-DK 127531 01 A1 (KAG).
- National Institutes of Health grant F32-AWD023945 (TMB).
- VESS Medtronic Resident Research Award (TMB).

## Supplementary Material

Supplemental data

Unedited blot and gel images

Supporting data values

## Version 1 11/24/2025

Electronic publication
