# Supplementary material for: TNF-α represses fibroblast to myofibroblast transition through the histone methyltransferase Setdb2
Source: JCI Insight. 2025 Nov 24;10(22):e190836. doi: 10.1172/jci.insight.190836 (PMC12643505; doi:10.1172/jci.insight.190836)
Supplement: Supplemental data [file jciinsight-10-190836-s135.pdf]

Figure S1:

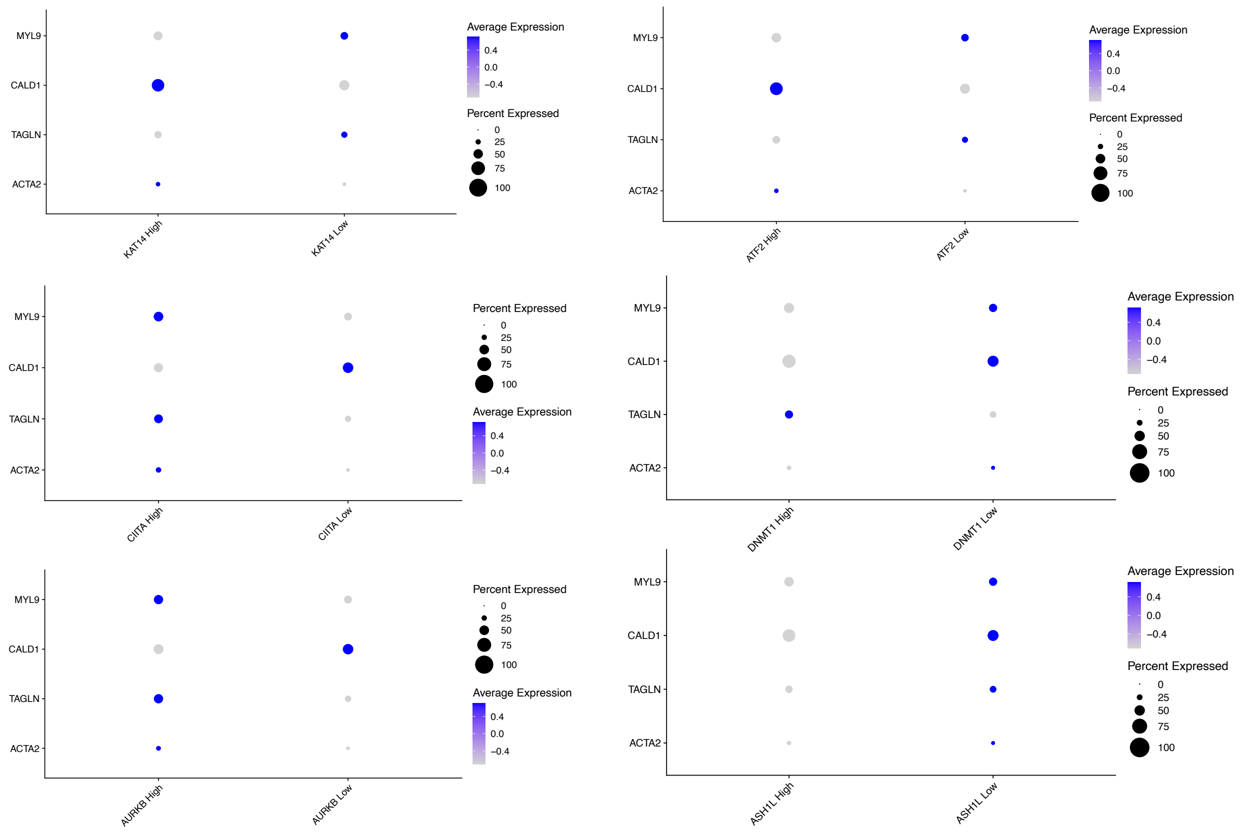

Dotplots detailing myofibroblast gene expression between human wound fibroblasts expressing high amounts of CMEs known to be upregulated by TNF-α.

Figure S2:

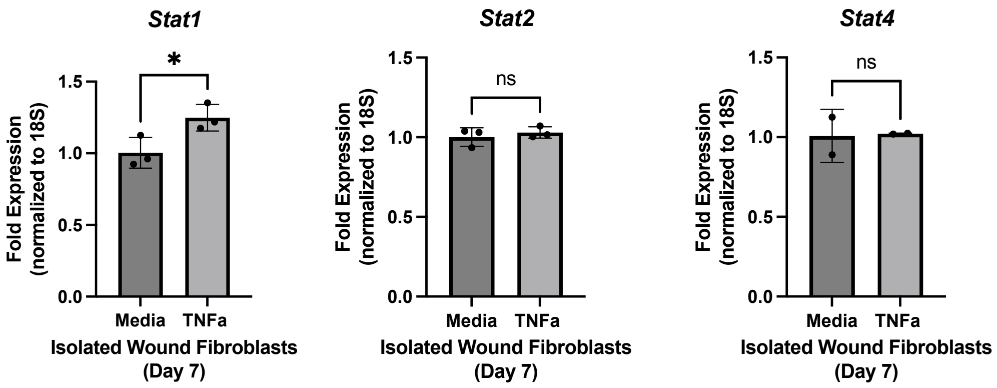

(A) Expression of *Stat1, 2,* and *4* at 6 hours following TNF-α stimulation. (N=4 mice/group, run in triplicate).
