# Supplementary material for: TNF-α represses fibroblast to myofibroblast transition through the histone methyltransferase Setdb2
Source: JCI Insight. 2025 Nov 24;10(22):e190836. doi: 10.1172/jci.insight.190836 (PMC12643505; doi:10.1172/jci.insight.190836)
Supplement: Unedited blot and gel images [file jciinsight-10-190836-s136.pdf]

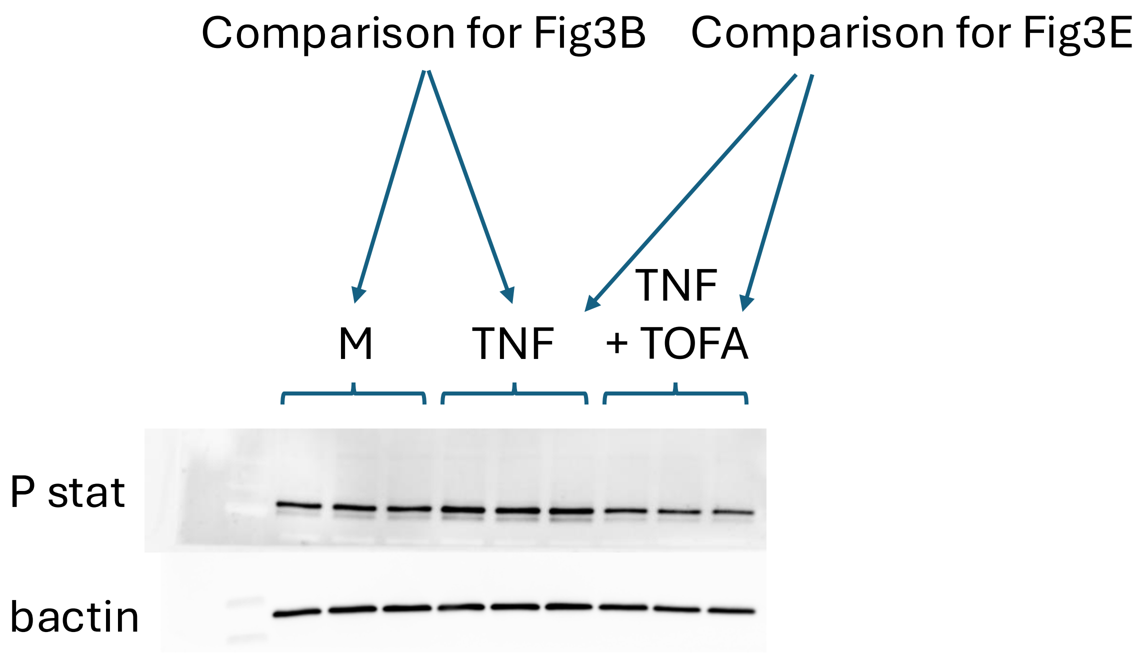
Comparison for Fig3B
Comparison for Fig3E
M
TNF
TNF
+ TOFA
P stat
bactin
